# Differential sensitivity of three forms of hippocampal synaptic potentiation to depotentiation

**DOI:** 10.1186/s13041-019-0451-6

**Published:** 2019-04-03

**Authors:** Pojeong Park, Thomas M. Sanderson, Zuner A. Bortolotto, John Georgiou, Min Zhuo, Bong-Kiun Kaang, Graham L. Collingridge

**Affiliations:** 1 Department of Biological Sciences and Brain and Cognitive Sciences, College of Natural Sciences, Seoul National University, Seoul, 151-746 Korea; 2 Department of Physiology, Faculty of Medicine, University of Toronto, 1 King’s College Circle, Toronto, Ontario M5S 1A8 Canada; 3 Lunenfeld-Tanenbaum Research Institute, Mount Sinai Hospital, Toronto, Ontario M5G 1X5 Canada; 4 Centre for Synaptic Plasticity, School of Physiology and Pharmacology and Neuroscience, University of Bristol, Dorothy Hodgkin Building, Whitson Street, Bristol, BS1 3NY UK

**Keywords:** Long-term potentiation, Depotentiation, Hippocampus

## Abstract

Theta-burst stimulation (TBS) induces short-term potentiation (STP) plus two types of transcriptionally-independent forms of long-term potentiation (LTP), termed LTP1 and LTP2. We have compared the susceptibility of these three types of synaptic plasticity to depotentiation, induced by low frequency stimulation (LFS; 2 Hz for 10 min) at the Schaffer collateral-commissural pathway in area CA1 of adult rat hippocampal slices. In interleaved experiments, STP and LTP were induced by three episodes of either compressed or spaced TBS (cTBS or sTBS). LFS had a more pronounced effect on the LTP induced by the cTBS. One traditional interpretation of these results is a difference in the time-dependent immunity against depotentiation. We suggest an alternative explanation: LFS rapidly reverses STP to reveal a slowly developing LTP. The cTBS protocol induces LTP1 that is moderately sensitive to depotentiation. The sTBS induces an additional component of LTP (LTP2) that is resistant to depotentiation.

**Electronic supplementary material:**

The online version of this article (10.1186/s13041-019-0451-6) contains supplementary material, which is available to authorized users.

## Main text

**Fig. 1:**
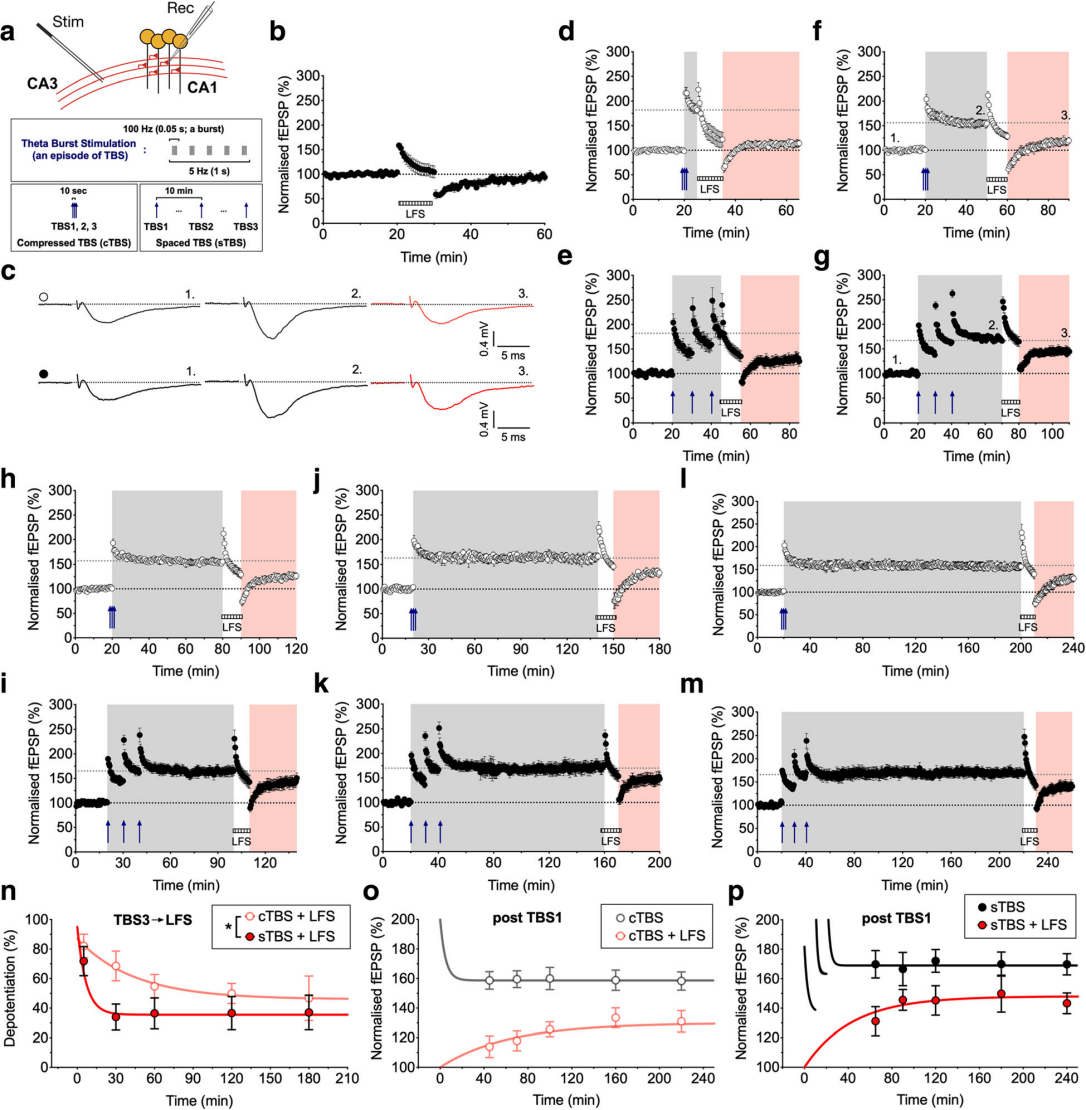
Distinct depotentiation of LTP induced by compressed versus spaced theta burst stimulation (TBS). **a** Schematic for extracellular recordings at Schaffer collateral-commissural CA1 synapses from adult rat hippocampus. The induction protocols for compressed (c) and spaced (s) TBS are summarized below. **b** The effects of LFS (2 Hz for 10 min) indicated by the hatched grey bar on naïve (i.e., unconditioned) synapses (*n* = 8). **c** Representative traces for baseline, LTP and depotentiation from experiments **f** and **g** (timing indicated by numbers). **d**-**m** The effects of LFS on cTBS- and sTBS-induced LTP after 5 min (**d** and **e**; *n* = 11 and 6), 30 min (**f** and **g**; *n* = 11 and 10), 60 min (**h** and **i**; *n* = 11 and 10), 120 min (**j** and **k**; *n* = 6 and 8) and 180 min (**l** and **m**; *n* = 10 and 7) after the last TBS. **n** Summary plot for the levels of depotentiation for cTBS and sTBS. The level of depotentiation is expresssed as the % reversal (from the potentiated response to the initial baseline) measured 30 min after the end of LFS, and plotted according to the time between TBS3 and LFS (TBS3 ➔ LFS). Regressions are statistically different (*p < 0.05). **o**-**p** Potentiation of synaptic responses following cTBS (**o**) or sTBS (**p**) for pathways receiving LFS (red) and their controls (black). The plots reveal the time course of development of LTP

N-methyl-D-aspartate receptor (NMDAR)-dependent synaptic potentiation is not a unitary process, but it can be divided into several temporally and mechanistically distinct components. Following a brief period of high-frequency stimulation followed by regular test pulses, there is a decaying phase, known as short-term potentiation (STP), and a persistent phase known as long-term potentiation (LTP). LTP can also be further subdivided into two components (termed LTP1 and LTP2) based on its sensitivity to inhibitors of protein kinase A (PKA), protein synthesis and calcium-permeable AMPA receptors (CP-AMPARs) (e.g., [1]). A single or compressed burst of high frequency stimulation, such as theta burst stimulation (TBS), induces LTP1 which is independent of these factors. Multiple spaced stimuli, with an inter-episode interval in the order of minutes, induces LTP of similar magnitude but a substantial component (i.e., LTP2) is sensitive to inhibitors of PKA, protein synthesis and CP-AMPARs. In the present study, we compared in interleaved experiments, LTP induced by a compressed (cTBS) and a spaced (sTBS) induction protocol, where the only difference was in the inter-episode interval (10 s vs. 10 min; Fig. 1a). Both protocols induced a decaying STP that stabilized into an LTP of similar magnitude (Additional file 1: Figure S1a).

The persistence of LTP can be disrupted by low-frequency stimulation (LFS) by a phenomenon known as depotentiation (e.g., [2–10]). In the absence of prior LTP, LFS (2 Hz for 10 min) induced a transient depression of synaptic responses that recovered to baseline within 30 min (*n* = 8; Fig. 1b). In contrast, when delivered after the induction of LTP, LFS invariably induced depotentiation of the synaptic response (Fig. 1c-n). We varied the timing between the induction of LTP and the delivery of LFS from between 5 and 180 min and quantified the level of depotentiation, 30 min from the end of the LFS train (Fig. 1d-n).

When LFS was delivered 5 min after the end of cTBS (Fig. 1d) or sTBS (Fig. 1e) the level of depotentiation was similar (82 ± 8% and 72 ± 10%, respectively). With a 30 min interval the level of depotentiation decreased to 69 ± 10% and 34 ± 9%, respectively (*p < 0.05). From 60 min onwards, the level of depotentiation described by a single exponential regression plateaued at 46 and 36%, respectively (*p* > 0.05). Comparison of the two plots revealed a significant difference over the time-course of the experiment (*p < 0.05; Fig. 1n). We also plotted the depotentiation data relative to the second (Additional file 1: Figure S1g) and first (Additional file 1: Figure S1h) bout of TBS. Although there was still a trend for a difference in the levels of depotentiation, this was not statistically significant.

An alternative way of interpreting these data is that LFS completely depotentiates STP, an hypothesis compatible with the extreme activity-dependence of decay of STP [11]. Consequently, what is observed is the gradual development of LTP. Consistent with this interpretation, previous work has shown that, in response to high frequency stimulation, LTP comprises an initial presynaptic change, that corresponds in time with STP, and a gradually developing postsynaptic change, as detected by alterations in sensitivity to the activation of AMPA receptors [12]. We performed paired-pulse experiments that confirmed there is a slowly decaying presynaptic STP in response to both cTBS and sTBS (Additional file 1: Figure S1b-f). To compare the overall development of potentiation after LFS, we plotted the size of the fEPSP as a function of time after the initial TBS. LTP developed slowly in response to both cTBS (Fig. 1o) and sTBS (Fig. 1p), but was considerably larger in the latter case. Time-matched comparisons of the extent of LTP, with and without LFS, revealed a statistically significant susceptibility of LTP to LFS for cTBS but not for sTBS (Additional file 1: Figure S1j-k). Therefore, these results can be interpreted by the co-existence of STP, that is highly sensitive to depotentiation, plus a time-invariant (over the course of these experiments) depotentiation of LTP1. The depotentiation of LTP induced by sTBS can be largely, if not exclusively, explained by the LTP1 component of the response, which comprises ~ 30–40% of the sTBS-induced LTP [13]. Therefore, LTP2 is highly resistant to depotentiation.

Synaptic potentiation at CA1 synapses is often considered as a unitary phenomenon. However, at least three mechanistically-distinct forms of NMDA receptor-dependent synaptic potentiation co-exist during the first few hours following its induction [1]. High frequency transmission (e.g., TBS) induces an initial STP, that is mediated by an increase in neurotransmitter release probability, followed by stable LTP, that is mediated, at least in part, by postsynaptic alterations. Our new paired-pulse facilitation data, presented in Additional file 1: Figure S1b-f, is consistent with this distinction. By comparing the ability of LFS to affect synaptic potentiation induced by cTBS and sTBS over a 3 h time interval we can propose the following: First, that STP, induced by either cTBS or sTBS, is the most sensitive form of synaptic plasticity to the effects of LFS. This sensitivity can be explained by the extreme activity-dependent decay of STP [11]. Second, that LTP1 is more sensitive than LTP2 to depotentiation. In this regard, it should be noted that at least a portion of the depotentiation of the LTP induced by a sTBS can be attributed to depotentiation of the LTP1 that is invariably induced along with LTP2. The most straightforward interpretation of our results is that STP is rapidly and fully depotentiated by LFS, LTP1 displays a moderate, potentially time-independent depotentiation and LTP2 is insensitive to depotentiation.

Our findings are consistent with the notion that resistance against depotentiation requires protein synthesis [9]. In other words, LTP2 is more resistant to depotentiation because of the nature of the potentiation induced, which likely involves morphological changes. In contrast, LTP1, which probably involves primarily post-translational modifications affecting AMPARs at the synapse [1], is more readily reversible. Indeed, both alterations in the single-channel conductance properties and the number of AMPARs at synapses can be rapidly and fully reversed by depotentiation [10]. In conclusion, three forms of synaptic potentiation co-exist but can be clearly distinguished on the basis of their sensitivity to depotentiation.

## Materials and methods

### Hippocampal slice preparation

Experiments were performed as described previously [13–15]. In brief, transverse hippocampal slices (400 μm) were prepared from male Sprague-Dawley rats (10–12 weeks of age). Animals were anesthetized with isoflurane and sacrificed by decapitation in accordance with Korea and UK animal legislation. The brain was then removed and placed in ice-cold slicing solution that contained (mM): 124 NaCl, 3 KCl, 26 NaHCO_3_, 1.25 NaH_2_PO_4_, 10 MgSO_4_, 10 D-glucose and 1 CaCl_2_, saturated with 95% O_2_ and 5% CO_2_. The hippocampi were rapidly isolated from the brain and sliced using a vibratome (Leica, VT1000S) while maintained in the slicing solution. The CA3 region was surgically removed to suppress the upstream neuronal excitability, and the slices were transferred to an incubation chamber that contained the recording solution (artificial cerebrospinal fluid, ACSF; mM): 124 NaCl, 3 KCl, 26 NaHCO_3_, 1.25 NaH_2_PO_4_, 2 MgSO_4_, 10 D-glucose and 2 CaCl_2_ (oxygenated with 95% O_2_ and 5% CO_2_). Slices were allowed to recover at 32–34 °C for 30 min, and then maintained at 26–28 °C for a minimum of 1 h before recordings were made.

### Field excitatory postsynaptic potential (fEPSP) recordings

The extracellular electrophysiology was performed in an interface type chamber maintained at 32 °C, and continuously perfused at 2 mL/min with the oxygenated ACSF. The slope of evoked fEPSPs (V/s) was measured in the CA1 region of hippocampal slices and bipolar stimulating electrodes were used at a constant intensity (0.1 ms pulse width) throughout the experiments. Signals were amplified using Axopatch 1D (Molecular Devices) and digitized with BNC-2110 (National Instruments) A/D board at a sampling rate of 20 kHz. Recordings were monitored and analyzed using WinLTP. Each experiment was conducted on slices from separate animals, so the n-value reflects both the number of slices and animals used.

Schaffer collateral-commissural fibres were stimulated to obtain the evoked synaptic responses, each at a constant frequency of 0.033 Hz. Following a stable baseline period of at least 20 min, LTP was induced using theta-burst stimulation (TBS) delivered at the same basal stimulus intensity. An episode of TBS comprises 5 bursts at 5 Hz, with each burst composed of 5 pulses at 100 Hz. For compressed (c) TBS induction of LTP, three TBS episodes were delivered with an inter-episode interval (IEI) of 10 s. For spaced (s) TBS, the same number of episodes were given with an IEI of 10 min. Representative sample traces are an average of 5 consecutive responses, collected from typical experiments (stimulus artifacts were blanked for clarity).

To measure the reversibility of LTP (i.e., depotentiation), low-frequency stimulation (LFS; 2 Hz for 10 min) was given at various time points (5, 30, 60, 120 and 180 min) following cTBS and sTBS. Often two-input experiments were performed to ensure recording stability. Since we found no difference between one and two-input studies, these results were pooled. In order to quantify depotentiation (%), the last 60 s of responses of LTP (pre-LFS) and the 60 s of responses obtained 30 min after the end of LFS were compared. If LFS had no effect on the response size then depotentiation would be considered to be 0% whereas if LFS restored the synaptic response to the pre-TBS baseline then depotentiation would be considered to be 100%. The actual values fell between these ranges.

### Statistical analysis

Data are presented as mean ± SEM (standard error of the mean). Responses were normalised to the baseline prior to LTP induction. Statistical significance was assessed using ANOVA with Bonferroni’s correction; the level of significance is denoted as follows: *p < 0.05, **p < 0.01 and ***p < 0.001. Regression curves are single exponentials and compared by extra sum-of-squares F-test. In Fig. 1o-p, regressions and time-matched data plots for LTP were obtained from Fig. 1l-m (except for the 220 and 240 min data points that were derived from Additional file 2: Figure S2).

## Additional files

Additional file 1: **Figure S1.** Differential sensitivity of STP, LTP1 and LTP2 to depotentiation. (a) Pooled data (*n* = 6) showing the effects on synaptic potentiation of a sTBS on one input followed 30 min later by a cTBS on a second independent input. Paired-pulse stimulation (50 ms intervals) was delivered throughout the experiments. (b) A plot of the paired-pulse ratio for the sTBS input. (c) Plot of paired-pulse ratio for the cTBS input. Note that there is a reduction in the paired-pulse ratio during STP, but not after LTP had stabilized. (d-f) Plot of the paired-pulse ratio as a function of time following either first (TBS1), second (TBS2) or the last TBS (TBS3). (g-h) Extent of depotentiation for cTBS and sTBS, plotted as a function of the time between TBS2 and LFS (g) and TBS1 and LFS (h). (i) Plot of the time course of synaptic potentiation induced by cTBS versus sTBS either in the presence or absence of LFS (pooled plots from Fig. 1o-p). (j) Individual data and statistical analysis showing the ability of LFS to effectively depotentiate cTBS-evoked synaptic potentiation at time-matched points. (k) In contrast, LFS had no significant effect when LTP, induced by sTBS, had reached a plateau. ***p < 0.001; **p < 0.01; *p < 0.05 versus control. (TIFF 1040 kb)

Additional file 2: **Figure S2.** Additional LTP experiments. (a) Pooled data (*n* = 7) showing the effects of cTBS monitored during the course of 5 h. (b) The same experiments except for sTBS used for LTP induction (*n* = 6). (TIFF 454 kb)

## Abbreviations

AMPAR: α-amino-3-hydroxy-5-methyl-4-isoxazolepropionate receptor
cTBS: Compressed TBS
LFS: Low-frequency stimulation
LTP: Long-term potentiation
NMDAR: N-methyl-D-aspartate receptor
PKA: Protein kinase A
sTBS: Spaced TBS
STP: Short-term potentiation
TBS: Theta-burst stimulation
