# Supplementary figures and images for: Differential sensitivity of three forms of hippocampal synaptic potentiation to depotentiation
Source: Mol Brain. 2019 Apr 3;12:30. doi: 10.1186/s13041-019-0451-6 (PMC6446328; doi:10.1186/s13041-019-0451-6)

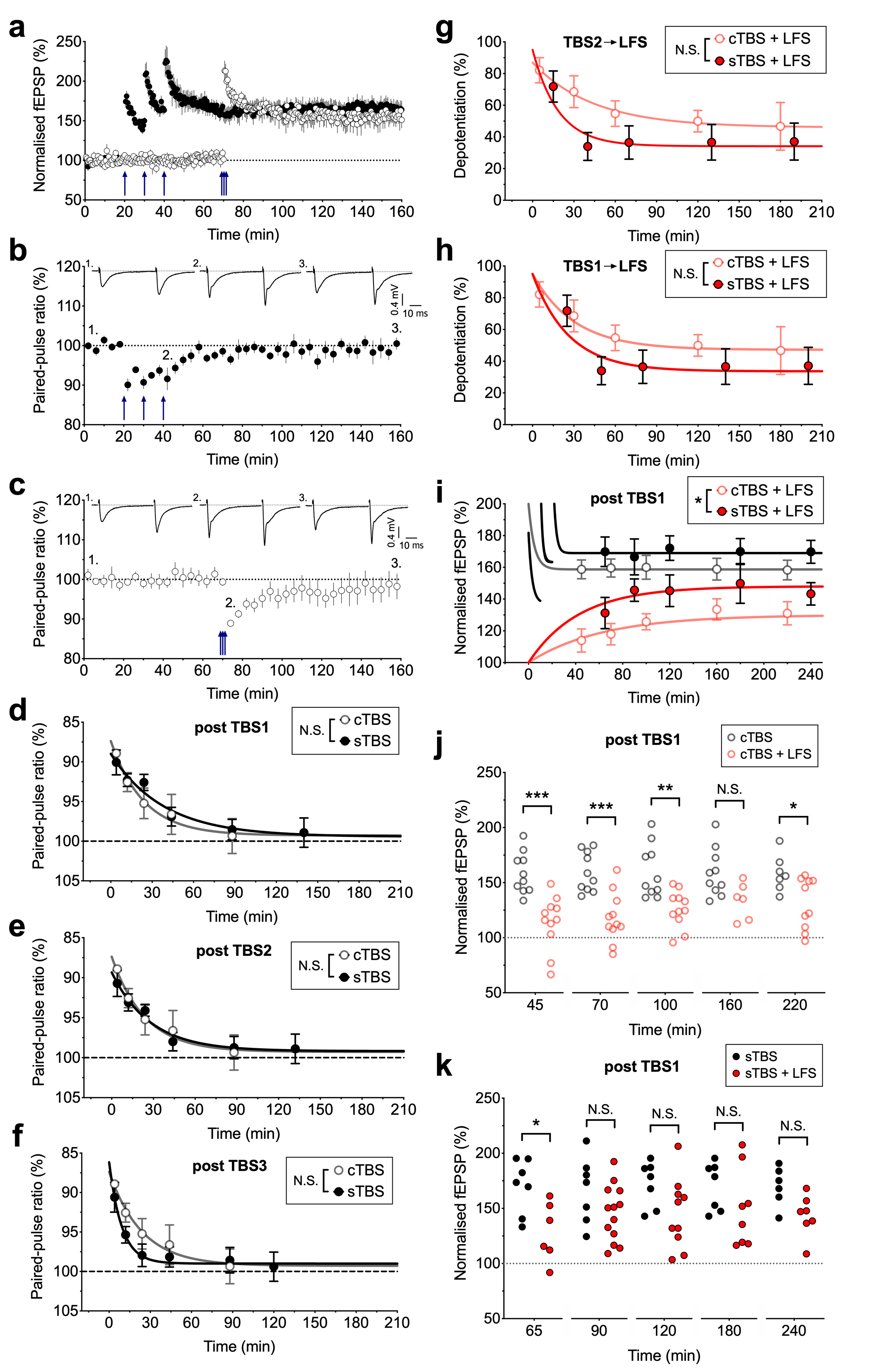

Supplement: Supplementary file 1 — Figure S1. Differential sensitivity of STP, LTP1 and LTP2 to depotentiation. (a) Pooled data (n = 6) showing the effects on synaptic potentiation of a sTBS on one input followed 30 min later by a cTBS on a second independent input. Paired-pulse stimulation (50 ms intervals) was delivered throughout the experiments. (b) A plot of the paired-pulse ratio for the sTBS input. (c) Plot of paired-pulse ratio for the cTBS input. Note that there is a reduction in the paired-pulse ratio during STP, but not after LTP had stabilized. (d-f) Plot of the paired-pulse ratio as a function of time following either first (TBS1), second (TBS2) or the last TBS (TBS3). (g-h) Extent of depotentiation for cTBS and sTBS, plotted as a function of the time between TBS2 and LFS (g) and TBS1 and LFS (h). (i) Plot of the time course of synaptic potentiation induced by cTBS versus sTBS either in the presence or absence of LFS (pooled plots from Fig. 1o-p). (j) Individual data and statistical analysis showing the ability of LFS to effectively depotentiate cTBS-evoked synaptic potentiation at time-matched points. (k) In contrast, LFS had no significant effect when LTP, induced by sTBS, had reached a plateau. ***p < 0.001; **p < 0.01; *p < 0.05 versus control. (TIFF 1040 kb) [file 13041_2019_451_MOESM1_ESM.tiff]

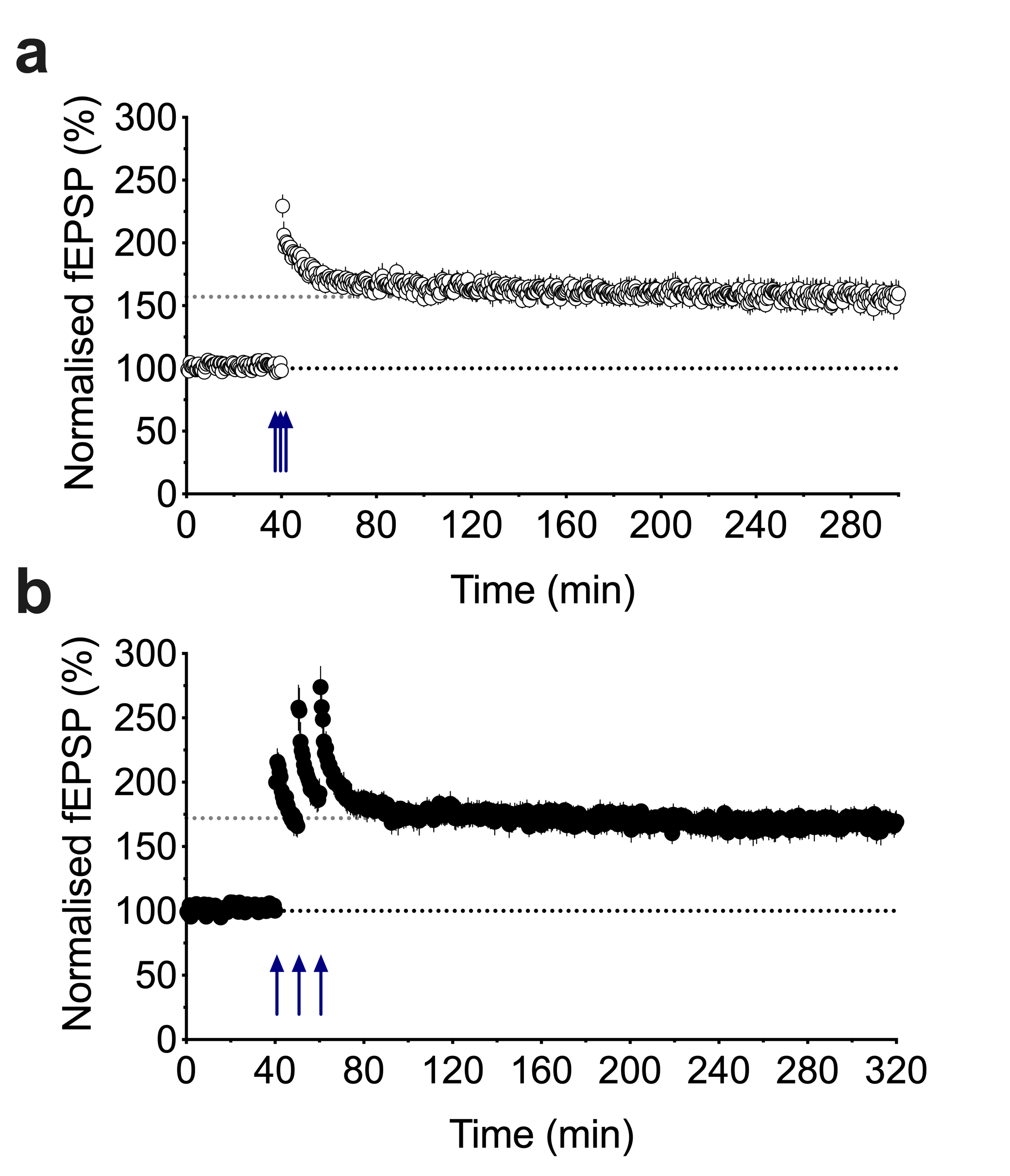

Supplement: Supplementary file 2 — Figure S2. Additional LTP experiments. (a) Pooled data (n = 7) showing the effects of cTBS monitored during the course of 5 h. (b) The same experiments except for sTBS used for LTP induction (n = 6). (TIFF 454 kb) [file 13041_2019_451_MOESM2_ESM.tiff]
